# Chronic migraine classification: one more attempt of optimization and criteria revision

**DOI:** 10.1007/s10194-012-0429-6

**Published:** 2012-04-07

**Authors:** Vera Osipova, Guzyal Tabeeva, Tatiana Voznesenskaya

**Affiliations:** Department of Neurology and Clinical Neurophysiology, First Sechenov Moscow State Medical University, Ul.Rossolimo, 11, 119021 Moscow, Russia

First of all we would like to thank Manzoni et al. for the deep and thoughtful discussion on chronic migraine (CM) classification [1]. All of us who deal with CM patients feel sharp need in universal and complete classification of this complicated and ambiguous disorder, especially taking into account the problem of medication overuse [2, 3]. Below, we present our opinion on the latest revision of the ICHD-2 for migraine suggested by the Italian team and propose new revision of CM diagnostic criteria for ICHD-3.

**Table:** Proposed revision of the ICHD-2 for migraine [1]

1.1 Migraine without aura
1.1.1 Infrequent migraine
1.1.2 Frequent migraine
1.1.3 Chronic migraine
1.1.3.1 With medication overuse
1.1.3.2 Without medication overuse
1.5 Complications of migraine
1.5.1 Transformed migraine
1.5.1.1 With medication overuse
1.5.1.2 Without medication overuse

The term “transformation” is known to mean the period when the usual course of migraine has begun to change (the main feature—gradual increase in attack frequency and the loss of typical migraine signs), but the patient did not yet reach the degree of “chronic M” (CM), i.e., does not comply with the new proposed diagnostic criteria of CM (ICHD-2R, 2006) [4, 5]. If we consider that the term “transformed M” (TM) is important and really has independent clinical and diagnostic value, we can keep it in the text of ICHD as the pre-stage of CM (with the diagnostic criteria proposed by Silberstein et al. or somehow modified) [6] (Option 1).

**Table:** Option 1

1.1 Migraine without aura
1.1.1 Infrequent episodic migraine
1.1.2 Frequent episodic migraine (without division below or with it)
1.1.2.1 With medication overuse
1.1.2.2 Without medication overuse
1.2 Migraine with aura
…
1.5 Complications of migraine
1.5.1 Transformed migraine
1.5.1.1 With medication overuse
1.5.1.2 Without medication overuse
1.5.2 Chronic migraine
1.5.2.1 With medication overuse
1.5.2.2 Without medication overuse
1.5.3 Status migrainosus

If we consider that the terms “transformed” and “chronic” reflect, to a considerable degree, similar if not the same course of M evolution they could be used as synonyms (since chronification is a terminal stage of transformation) [7]. In this case, the term “chronic” reads more literate and could be used in the text of the ICHD instead of the term TM.

In this case it is important to note: since the term CM “absorbs” the term TM, diagnostic criteria of CM should also “absorb” all or some of the most prominent diagnostic criteria of TM [6]. Below we present our version of such combined criteria.

Chronic migraine (ICHD-3) Osipova, Tabeeva, Voznesenskaya (2012) [5, 6]

**Table:** 

Diagnostic criteria
A. History of episodic migraine meeting criteria for migraine without (with?) aura (possibly) relieved by triptan(s) or ergots
B. History of headache transformation with increasing headache frequency and decreasing migrainous features severity
C. For >3 latest month headache is present > 15 d/month with following characteristics
1. > 5 attacks/month, fulfilling criteria for migraine without (with?) aura
2. On >8 days/month patient has continuous mild to moderate headache with MO-like, TTH-like or mixed characteristics not completely fulfilling criteria for MO and TTH^a^
D. With or without medication overuse (triptans/ergots/complex analgesics)
E. Headache is not attributed to another disorder

^a^We see an inaccuracy in the third criteria of CM in the latest ICHD-2R version: “On > 8 d/month for > 3 month, headache fulfills criteria for MO and/or treated and relieved by triptan(s) or ergot”. Clinical experience shows that in the majority of CM patients these “background” interim headaches are far from fulfilling MO criteria. Moreover, the patients can use not only triptans/ergots but also complex analgesics to relieve headache

It is also reasonable that the possibility of chronification as the main variant of migraine evolution/complications could be mentioned in ICHD only once, either under 1 or under 1.5 (Options 2 and 3).

**Table:** Option 2

1.1. Migraine without aura
1.1.1. Episodic migraine (without division below or with it)
1.1.1.1. Infrequent episodic migraine
1.1.1.2. Frequent episodic migraine (without division below or with it)
1.1.1.2.1. With medication overuse
1.1.1.2.2. Without medication overuse
1.1.2 Chronic migraine
1.1.2.1. With medication overuse
1.1.2.2. Without medication overuse
1.5 Complications of migraine
1.5.1 Status migrainosus, etc

**Table:** Option 3

1.1 Migraine without aura
1.1.1. Infrequent episodic migraine
1.1.2. Frequent episodic migraine (without division below or with it)
1.1.2.1. With medication overuse
1.1.2.2. Without medication overuse
1.2. Migraine with aura
…
1.5. Complications of migraine
1.5.1 Chronic migraine (with new diagnostic criteria combining both CM and TM features)
1.5.1.1 With medication overuse
1.5.1.2 Without medication overuse
1.5.2. Status migrainosus

Any of these options could be chosen (we consider Option 3 the most appropriate) but the diagnostic criteria of TM should by all means be taken into account and added to CM criteria as proposed above. Although from our point of view the proposed criteria completely reflect the clinical essence and nature of CM, the contribution of the native English speaker in terms of linguistic editing could be needed. We believe that each brick in a stone-work serves to speed up the construction of the ICHD-3.
